# Carbon Molecular Sieve Membranes from Acenaphthenequinone–Biphenyl Polymer; Synthesis, Characterization, and Effect on Gas Separation and Transport Properties

**DOI:** 10.3390/polym17040541

**Published:** 2025-02-19

**Authors:** Jesús Ortiz-Espinoza, Olivia Hernández-Cruz, Mikhail Zolotukhin, F. Alberto Ruiz-Treviño, María Isabel Loría-Bastarrachea, Manuel Aguilar-Vega

**Affiliations:** 1 Materials Science, Membranes Laboratory, Yucatan Scientific Research Center, Calle 43 x 32 and 34, Chuburná de Hidalgo, Mérida 97205, Yucatán, Mexico; mallas.moleculares@cicy.mx (J.O.-E.); marisa@cicy.mx (M.I.L.-B.); 2 Institute of Materials Research, National Autonomous University of Mexico, Apartado Postal 70-360, CU Coyoacán, Ciudad de México 97205, Mexico; hecrol9@gmail.com; 3 Department of Engineering and Chemical Science, Iberoamerican University, Prol. Paseo de la Reforma No. 880, Ciudad de México 01219, Mexico; 1frankalbert@gmail.com

**Keywords:** acenaphthenequinone, carbon molecular sieve membranes, superacid catalyzed polymerization, crystallite thickness stacking, gas sorption, gas permeability, O_2_/N_2_ separation

## Abstract

A rigid, high temperature-resistant aromatic polymer, poly(1,1′-biphenyl)-6,8a-dihydroacenaphthylene-1(2H)-one (BDA) comprising acenaphthenequinone and biphenyl was successfully synthesized by superacid catalyzed polymerization. BDA has a high decomposition temperature (T_d_ = 520 °C) that renders it a viable candidate for carbon molecular sieve membranes (CMSM) formation. BDA precursor pyrolysis at 600 °C (BDA-P600) leads to a carbon turbostratic structure formation with graphene-like amorphous strands in a matrix with micropores and ultramicropores, resulting in a carbon structure with higher diffusion and higher selectivity than dense BDA. When the BDA pyrolysis temperature is raised to 700 °C (BDA-P700), the average stacking number of carbon layers *N* increases, along with an increase in the crystallite thickness stacking *L_c_*, and layer plane size *L_a_*, leading to a more compact structure. Pure gas permeability coefficients *P* are between 3 and 5 times larger for BDA-P600 compared to the BDA precursors. On the other hand, there is a *P* decrease between 10 and 50% for O_2_ and CO_2_ between CMSM BDA-P600 and BDA-P700, while the large kinetic diameter gases N_2_ and CH_4_ show a large decrease in permeability of 44 and 67%, respectively. It was found that the BDA-P700 WAXD results show the emergence of a new peak at 2θ = 43.6° (2.1 Å), which effectively hinders the diffusion of gases such O_2_, N_2_, and CH_4_. This behavior has been attributed to the formation of new micropores that become increasingly compact at higher pyrolysis temperatures. As a result, the CMSM derived from BDA precursors pyrolyzed at 700 °C (BDA-P700) show exceptional O_2_/N_2_ gas separation performance, significantly surpassing baseline trade-off limits.

## 1. Introduction

Membrane gas separation is becoming an increasingly prominent area of research as an energy-efficient and eco-friendly alternative to conventional separation technologies [1,2]. Polymeric membranes are widely applied due to their cost-effectiveness and processability [3]. However, these membranes face a trade-off between permeability and selectivity, which limits their potential for industrial applications [4].

Carbon molecular sieve (CMS) membranes have recently attracted considerable interest as high-performance materials for gas separation, offering a distinctive combination of exceptional permeability, selectivity, and operational resilience [5]. CMS membrane properties are related to the precursor polymer selection which affects membrane microstructure, transport characteristics, and overall separation efficiency. Among the various precursor candidates, those with simple yet strategically designed molecular structures are particularly appealing due to their synthetic simplicity, cost-effectiveness, and scalability. Some precursors used in the formation of CMS are phenolic resins (PR) [6], polyimides (PIs) [7], poly (ether imide) (PEI) [8], and polybenzimidazole (PBI) [9], as well as cellulose and its derivatives [10]. Polyimides, due to their excellent mechanical and thermal properties and rigid structure, stand out as an exceptional precursor for the formation of CMS membranes. PI polymers are well-known for their high thermal stability, withstanding pyrolysis at extremely high temperatures, forming CMSM with enhanced gas transport properties. Particularly, PIs based on 6FDA due to the presence of a -CF_3_ group show a high gas separation performance [11]. C:P Hu et al. studied how gas separation performance was affected in CMSM by differences in the chain rigidity and free volume that led to differences in the microstructure of the final membrane [12]. K. Li et al. determined the effect on CMSM microstructure and pore formation after the pyrolysis of high free volume PIM-PIs based in different isomers; they found that a high surface area and large micropores improved their gas permeability and selectivity [13]. O. Sanyal et al. [14] indicated that increasing the final pyrolysis temperature tightens the micropores and ultramicropores, which increases diffusion selectivity via a gas diffusion coefficient decrease due to the compacted micropore sizes. They also indicated that reduced micropores tend to lower the gas solubility coefficients. The overall result is a decrease in permeability as the pyrolysis temperature increases, with an increase in gas selectivity.

In previous studies carried out in our research group, the synthesis of multiring aromatic copolymers based on acenaphthenequinone and isatin using superacid catalyzed click chemistry was reported [15]. These multiring, highly aromatic, high temperature-resistant copolymers were pyrolyzed at 600 °C to form CMSM that present at least a one order of magnitude increase in gas permeability. The copolymers also showed at least two times the increase in selectivity with respect to those presented by the precursor base multiring copolymers. The synthesis of highly aromatic rigid poly(oxo-biphenylene-isatin), POBI, polymers using superacid click chemistry have also been reported [16]. Thin film membranes prepared from POBI polymers exhibited a systematic increase in pure gas transport properties attributable to the incorporation of lateral phenyl–fluorine groups. CMS membranes obtained from POBI’s pyrolysis at 600 °C showed, in some cases, a 10- fold increase in gas permeability compared to the POBI precursor. The bulky fluorine moieties increased *P*(CO_2_) six times for POBI’s, and the separation factor for the gas pair CO_2_/CH_4_ increased from 10.7 to 47.

**Figure 1:**
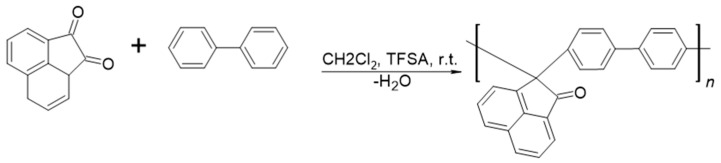
Schematic polymerization of poly (biphenyl-6, 8-dihydroacenaphthalenyl.-1-ona) (BDA).

In this study, we explore a highly aromatic, high temperature poly (biphenyl-6, 8-dihydroacenaphthalenyl-1-ona) (BDA) obtained by superacid click chemistry from acenaphthenoquinone and biphenyl moieties. BDA’s thermal, chemical, and gas transport properties were characterized. Figure 1 illustrates the general synthesis reaction for poly (biphenyl-6, 8-dihydroacenaphthalenyl-1-ona) (BDA). Firstly, BDA was employed for dense membrane preparation. Secondly, BDA was used as a precursor to form CMSM pyrolyzed at two final temperatures, 600 °C and 700 °C. The changes in the carbonaceous graphene-like structure, carbon strata stacking, and gas transport properties due to the final pyrolysis temperature were systematically studied. The structural changes in CMS membranes from BDA according to the pyrolysis temperature are evaluated using Fourier transform infrared spectroscopy (FTIR) and thermal analysis (TGA). Additionally, wide angle X-ray diffraction (WAXD), Raman spectroscopy, X-ray photoelectron spectroscopy (XPS), and CO_2_ adsorption isotherms are used to gain further insights into the final CMS membrane carbonaceous and turbostratic structure. Finally, the effect of pyrolysis temperature on CMSM pure gas transport properties at different temperatures is assessed.

## 2. Materials and Methods

### 2.1. Materials

Commercially available materials and solvents were purchased from Aldrich Chemical Co (St. Louis, MO, USA). Aceanthrenequinone was recrystallized from 1, 2-dichlorobenzene using charcoal. Biphenyl was used without further purification. Trifluoromethanesulfonic acid (98%, TFSA), trifluoroacetic acid (≥99%, TFA), methanol (technical grade, MeOH), methylene chloride (CH_2_Cl_2_), and 1-methyl-2-pyrrolidone (NMP) were provided by Aldrich Chemical Co. All solvents and superacids were distilled prior to use [17].

Polymer Synthesis: A solution was prepared by combining 0.4591 g (2.52 mmol) of acenaphthenequinone, 0.2775 g (1.8 mmol) of biphenyl, 0.7 mL of methylene chloride, and 1 mL of TFA in a 5 mL Erlenmeyer flask under a nitrogen atmosphere. Subsequently, the reaction mixture was placed into an ice bath and 1.28 mL of triflic acid (TFSA) was added. Following a five-minute period, the ice bath was removed, and the stirring continued at room temperature for six hours. After this time, 2 mL of TFA:CH_2_Cl_2_ in a 1:1 ratio was added as a solvent to obtain a homogeneous mixture before precipitation. The polymer was precipitated into methanol, filtered, washed with hot methanol, and dried. The resulting polymer (0.5927 g, 99%) exhibited an inherent viscosity of 1.09 dL g^−1^ in NMP.

### 2.2. BDA Polymer Characterization

BDA NMR spectra were obtained on a Bruker Avance Spectrometer (Zurich, Switzerland) operating at 400.13 MHz for ^1^H NMR and 100 MHz for ^13^C-NMR. FT-IR spectra were acquired on a Nicolet IS10 Thermo Scientific spectrometer (Rochester, NY, USA). Inherent viscosities in 0.2 wt% polymer solutions were measured at 25 °C using an Ubbelohde viscometer (No. 50) (Ace glass Inc. Vineland, NJ, USA) and 1-methyl-2-pyrrolidinone (NMP) as the solvent. BDA solubility tests were performed in dichloromethane (DCM), chloroform (CHCl_3_), 1,1,2,2-tetrachloroethane (TCE), tetrahydrofuran (THF), N,N-Dimethylacetamide (DMAc), N,N-dimethylformamide (DMF), dimethyl sulfoxide (DMSO), sulfuric acid (H_2_SO_4_), cyclohexane, 1,4-dioxane, and 1-methyl-2-pyrrolidone (NMP). Thermogravimetric analysis (TGA) was carried out in a TGA-8000 (Perkin Elmer, Springfield, IL, USA) thermobalance, at a heating rate of 10 °C min^−1^ between 50 and 800 °C under a nitrogen atmosphere.

### 2.3. Dense Membrane Formation

Polymer dense membranes were prepared as thin non-porous films by the solvent evaporation method where the membrane was cast on an aluminum surface using chloroform solutions containing 5 wt% polymer. Finally, the dense membrane was removed from the aluminum and subjected to vacuum drying for 24 h at 120 °C, to ensure that all residual solvent was eliminated. The thickness of the membrane was found to be 75 ± 2 µm as measured with a Digimatic micrometer (Toyo) (Cincinnati, OH, USA) (IDC-112B-5). Density was determined at 23 °C in a density gradient column using a Techne two-column Density Gradient Column (Techne Corp, Princeton, NJ, USA). The columns were prepared from calcium nitrate Ca(NO_3_)_2_ solutions. Density measurements were performed according to the standard procedure outlined in ASTM D1505-68 [18]. The fractional free volume (FFV) was estimated as $FFV=\frac{v-1.3v_{W}}{v}$, where ν is the specific volume and ν_w_ is the Van der Waals volume, which was obtained through Bondi’s group contribution method [19].

### 2.4. Carbon Molecular Sieve (CMS) Membranes

BDA dense membranes were subjected to pyrolysis in an argon atmosphere, at two different final temperatures of 600 and 700 °C. Initially, BDA dense membranes were cut into square samples measuring one inch on each side and weighed, before being placed inside a quartz tube. Subsequently, the tube was placed within a three-zone furnace (Lindberg/Blue, model STF55346C-1) (Asheville, NC, USA). The pyrolysis protocol followed has been previously reported in the literature [20]. A standard protocol, comprising four stages for each final pyrolysis temperature, was employed. For BDA pyrolysis at 600 °C (BDA-P600), initially the temperature increased from 25 to 250 °C at 13.3 °C/min. In the second heating stage, from 250 to 585 °C, the temperature ramp increased at 3.85 °C/min. Next, the temperature was reduced to 0.25 °C/min between 585 °C and 600 °C. In the fourth step, a 2 h dwell time at 600 °C was followed by cooling down the sample to room temperature. For BDA pyrolysis at 700 °C (BDA-P700), the protocol involved the same initial heating steps from 25 to 250 °C, at 13.3 °C/min; then, the temperature increase between 250 and 685 °C was performed at 3.85 °C/min. Finally, from 685 °C to 700 °C, the temperature increased at 0.25 °C/min, and the pyrolyzed samples were allowed a 2 h dwell time at 700 °C. Thereafter, they were allowed to cool down to room temperature. The use of this pyrolytic protocol typically results in carbon molecular sieve membranes (CMSM), formed by carbon turbostratic structures [21]. Finally, the BDA pyrolyzed membranes BDA-P600 and BDA-P700 were stored prior to pure gas permeability testing.

### 2.5. CMS Membrane Characterization

Raman spectra for BDA-P600 and BDA-P700 were obtained using a confocal Raman InVia microscope (West Dundee, IL, USA) with a 50X objective at a 633 nm wavelength, with a 10 s exposure time. Wide angle X-ray diffraction (WAXD) spectra were conducted on a Bruker D2 Phaser X-ray diffractometer (Burlingon, ON, Canada) (30 KV and 10 mA) with Cu K-α (λ = 0.154184 nm) radiation at a scanning rate of 2 min^−1^ from 5 to 70° 2θ. The *d*-spacing was determined by the equation *d* = λ/2sinθ, where θ is the X-ray diffraction angle. X-ray Photoelectron Spectroscopy (XPS) for both BDA-P600 and BDA-P700 membranes was performed on a Thermo Fisher Scientific K-Alpha XPS spectrometer (Rochester, NY, USA). An Al-Kα X-ray source at a 0.1 eV accelerating voltage was used with a 50 eV energy pass. Surface area and pore size for BDA-P600 and BDA-P700 CMS membranes were determined using an Anton Paar Quantachrome model Nova Touch LX2 (Boynton Beach, FL, USA) surface area and pore size analyzer with CO_2_ adsorption–desorption isotherms at 273 K via non-local density functional theory (NLDFT) analysis. The samples were outgassed at 473 K for 10 h to remove moisture content.

### 2.6. Pure Gas Transport Properties

Gas transport properties for BDA dense membranes, BDA-P600, and BDA-P700 were determined in a constant volume gas permeation cell built in our laboratories, which has been described in detail previously [22]. The pure gas permeability and apparent diffusion coefficients for five different pure gases, helium (He), oxygen (O_2_), nitrogen (N_2_), methane (CH_4_), and carbon dioxide (CO_2_), were determined using gases with a purity exceeding 99.99%, acquired from Praxair Corp (Mérida, Yucatán, México). The pure gas permeability coefficients (*P*) were ascertained under steady-state conditions at 35 °C and 2 atm upstream pressure, employing Equation (1):

(1) $$P=D\;*\;S=\left(\frac{273.15}{76}\right)*\left(\frac{VL}{ATp_{0}}\right)*{\left(\frac{dp}{dt}\right)}_{ss}$$

The gas permeability coefficient, *P*, is reported in Barrer $\left(1Barrer=1\times 10^{10}\left[\left(cc(STP\right)cm/\left({cm}^{2}\cdot s\cdot cmHg\right)\right]\right)$, while the downstream chamber volume, V, is expressed in cm^3^; *p*_0_ is the upstream gas pressure (cm Hg), *A* is the effective membrane area (cm^2^), *L* is the membrane thickness (cm), *T* is the operating temperature (K), and ${\left(\frac{dp}{dt}\right)}_{ss}$ is the steady-state pressure increase through the membrane (mmHg/s). The apparent diffusion coefficient (D, in cm^2^/s) of all membranes was calculated using the time-lag method (D = *l*^2^/6θ, where θ is the time-lag and *l* is the membrane thickness). The apparent solubility coefficient S (cm^3^ (STP)/(cm^3^ cm Hg)) (or apparent gas sorption, *S*, for CMSM) was calculated using the ratio between the *P* and *D* coefficients, $\underline{S}=P/D$. 

## 3. Results and Discussion

### 3.1. BDA Polymer Synthesis

**Figure 2:**
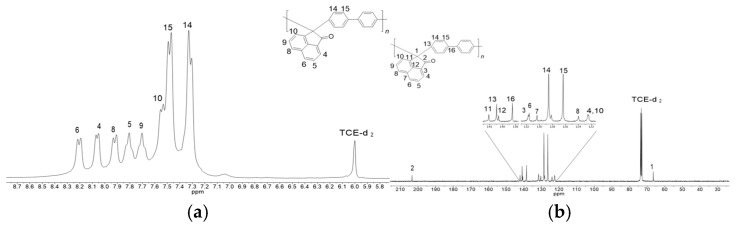
BDA ^1^H NMR (**a**) and ^13^C NMR (**b**) spectra in TCE-d_2_.

The dye, pharmaceutical, and pesticide industries widely use acenaphthenequinone, a quinone-type compound. Some studies have shown that acenaphthenequinone and some of its derivatives exhibit biological activity such as bactericidal, antihypoxic, and antifungal [23]. Acenaphthenequinone additionally produces superelectrophilic species in trifluoromethane sulfonic acid (TFSA) which react with moderate deactivated arenes in good yields [24]. In 2004, Zolotukhin et al. initially documented the synthesis of polymers based on acenaphthenequinone using a variety of polyaromatic compounds promoting the synthesis of poly(arylene oxindole) structures. This approach entailed the direct synthesis of unsubstituted biphenyl via an electrophilic substitution reaction within a polymeric synthesis context [25]. It later came to light that acidity levels influence the polymerization reaction, revealing that a medium acidity level (H0 less than −12, based on the Hammett scale) is appropriate for synthesizing this polymer within the range of medium molecular weight (0.40 dL g^−1^). A combination of TFSA and methanesulfonic acid (MSA) was determined to be the most appropriate for achieving this acidity [26]. On the other hand, it is well known that the molecular weight of a polymer directly impacts the physicochemical properties of the material. Research on superacid-catalyzed polymerization revealed that monomer concentration not only enhances the polymerization rate, often accelerating reaction kinetics, but also dramatically affects the molecular weight of the polymer, significantly increasing it [27,28,29]. In this sense, a 3:1 ratio of acenaphthenoquinone to biphenyl was explored, resulting in the synthesis of a polymer with a high inherent viscosity of 1.13 dL/g (measured at 25 °C in NMP at 0.5 g/dL), a density of 1.196 g/cm^3^, and a fractional free volume (FFV) of 0.18. BDA ^1^H NMR and ^13^C NMR polymer spectra are presented in Figure 2 as a reference. The spectra were acquired with deuterated tetrachloroethane (TCE-d_2_) as the solvent. The signals from the aromatic protons, observed between 7.25 and 8.25 ppm and corresponding to the BDA polymer, are presented in Figure 2a. Sharp doublets at 7.43 and 7.31 ppm indicate precise para-substitution in the biphenyl moieties at the main chain. Figure 2b displays the ^13^C NMR spectrum of the obtained BDA polymer, showing all 16 expected resonances, thus confirming the chemical structure and para-substitution pattern in the polymer through signal assignment.

### 3.2. BDA Polymer Solubility in Organic Solvents

Table 1 presents BDA polymer solubility in common organic solvents. In line with expectations based on its highly aromatic structure, BDA polymer was soluble only in sym-tetrachloroethane and 1-methyl-2-pyrrolidone. This finding aligns with previous reports in the literature where highly aromatic structures present low solubility due to the stacking of the aromatic rings that inhibit solvent access [30].

### 3.3. FTIR Spectrum

**Figure 3:**
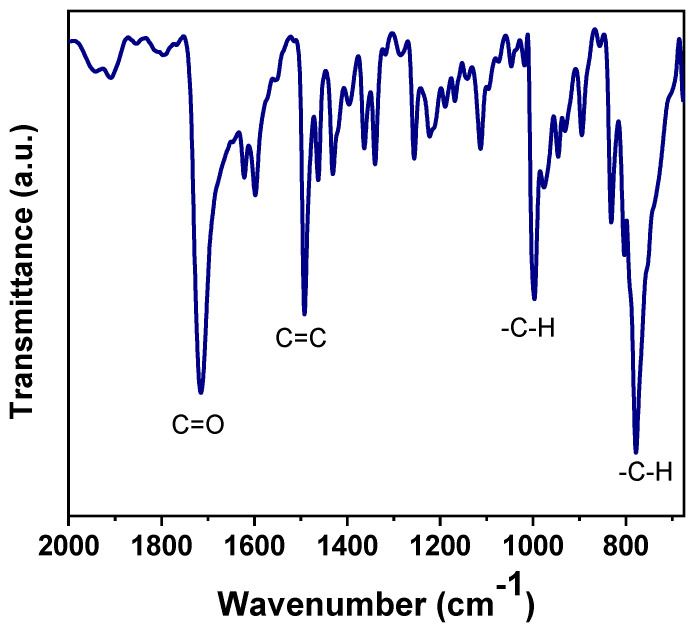
FTIR-ATR spectrum of BDA polymer.

BDA polymer structure was confirmed by FT-IR spectroscopic analysis. Figure 3 shows the BDA polymer membrane FTIR spectrum, which reveals several characteristic peaks and signals. The intense band at 1486 cm^−1^ for C=C stretching of the benzene ring is observed, as is the C=O signal at 1715 cm^−1^ associated with the ketonic group on the main structure [31]. Benzene C-H stretching is observed at 990 and 760 cm^−1^. These peaks confirm the BDA polymer final structure.

### 3.4. Thermal Properties

**Figure 4:**
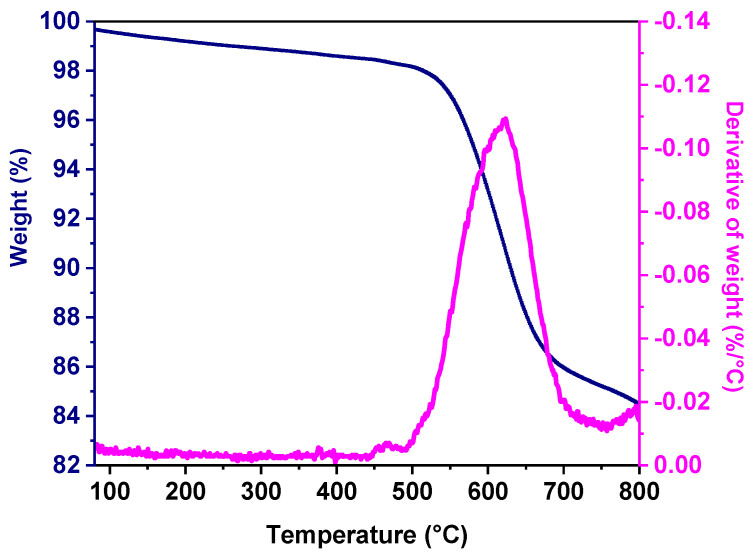
BDA polymer thermogravimetric analysis (TGA) weight loss curve (blue solid line) and thermogram derivative (magenta solid line).

Figure 4 illustrates a thermogram for BDA decomposition that, given its highly aromatic structure, shows high thermal resistance. The onset of decomposition starts at 515 °C, while a sharp decline in weight occurs up to 680 °C, followed by a levelling up at 700 °C. There is a small weight loss between 700 °C and 800 °C. Given BDA’s high thermal resistance there is a char yield at 800 °C of 85 wt%, an indication that the carbonaceous structure is preserved after pyrolysis under a nitrogen atmosphere. Based on the thermogravimetric analysis, the pyrolysis temperatures of 600 °C, the middle part of the decomposition, and 700 °C, where the decomposition temperature levels up, were chosen for the CMS membranes preparation.

### 3.5. Wide Angle X-Ray Diffraction (WAXD)

**Figure 5:**
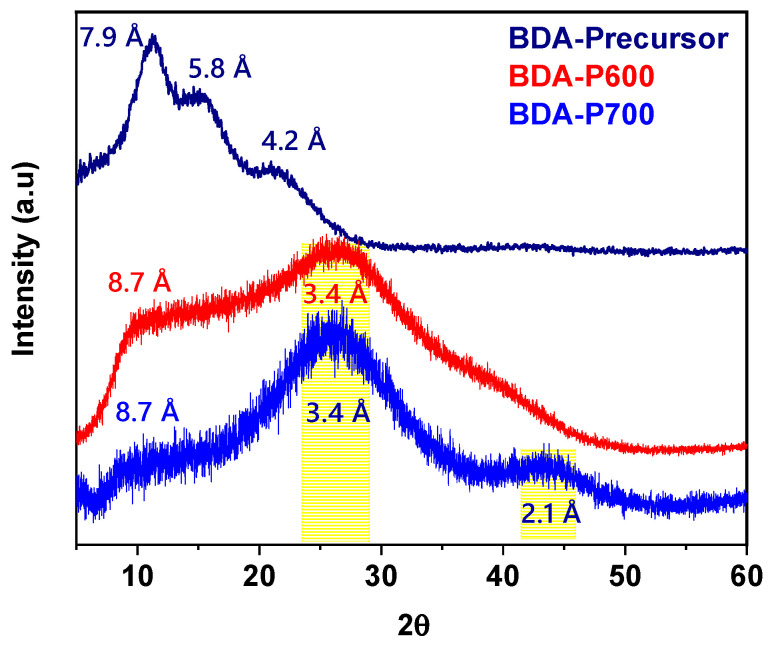
WAXD from BDA Precursor, BDA-P600, and BDA-P700 CMSM pyrolyzed to 600 and 700 °C, respectively.

Figure 5 illustrates the normalized wide-angle X-ray diffraction (WAXD) scattering patterns for the BDA polymer dense membrane in the range of 4 to 60° 2θ. The maximum peaks, which are related to the average intersegmental spacing (*d*-spacing) between chains, were calculated using Bragg’s equation. In the case of the BDA precursor, three peaks are observed, situated at 2θ = 11.3°, 15.2°, and 21.6°. The initial two peaks are ascribed to the primary interchain spacing (*d*-spacing at 7.9 Å and 5.8 Å) within the amorphous domain, whereas the subsequent peak, at 4.2 Å, can be attributed to the interchain spacing of the π-π stacking of aromatic rings as reported in the literature [32]. Figure 5 also presents the normalized WAXD patterns obtained for CMSM, BDA-P600, and BDA-P700. Broad diffraction amorphous peaks are observed in both BDA-P600 and BDA-P700 membranes. BDA-P600 and BDA-P700 exhibit two prominent amorphous halos at 2θ = 10.1° and 26.2°, reflecting their amorphous nature and the presence of micropores and ultramicropores in their structure. The pyrolysis process promotes the development of ultramicropores compared to the precursor membrane. Maxima for BDA-P600 and BDA-P700-CMSM WAXD spectra have been resolved by deconvolution using a model based on a set of Gaussian–Lorentzian functions (Figure S1). Additionally, the deconvolution results for the BDA precursor, along with those for BDA-P600 and BDA-P700 CMSM, are summarized in Table 2.

After pyrolysis at 700 °C, a new peak emerged at 2θ = 41.3°, corresponding to an interchain distance of 2.1 Å [33]. The broad peaks observed at diffraction angles of 2θ = 26.2° and 41.3° indicate that BDA-P600 and BDA-P700 possess an amorphous carbonaceous structure with graphitic-like crystallites stacked similarly to disordered graphite lattices [34]. These peaks correspond to the (002) and (100) planes of the lattice, respectively. Structural parameters, including the apparent layer plane length (*L_a_*), the apparent crystallite thickness (*L_c_*), and the average interlayer spacing (*d*-spacing), were determined for BDA-P600 and BDA-P700 using Bragg’s law and Scherrer’s equation [35], as summarized in Table 3.

As the pyrolysis temperature increased from 600 to 700 °C, the average graphitic crystallite interlayer spacing (*d*002) decreased, indicating a denser crystallite packing. The *d*002 value (3.42 Å) for BDA-P700 membranes is smaller than the molecular kinetic diameter of O_2_ (3.46 Å), N_2_ (3.64 Å), and CH_4_ (3.8 Å) gases. This implies that the membrane would effectively prevent the diffusion of these gases, as will be discussed in the following section. Moreover, the full width at half maximum (FWHM) of the (002) peak exhibited a reduction with increasing carbonization temperature, accompanied by an increase in the estimated crystallite stacking thickness (*L_c_*) and layer plane size (*L_a_*). This suggests the development of more ordered graphitic structures, a phenomenon that aligns with observations made by Xu et al. [33].

CMSM have slit-like nanoscale pores with a bimodal size distribution: larger micropores (7–20 Å) act as sorption sites and influence diffusion, while smaller ultramicropores (<7 Å) provide molecular sieving [36]. The distribution and size of the ultramicropores were determined using CO_2_ gas sorption at 0 °C. Figure S2a,b illustrates the CO_2_ adsorption isotherms of the BDA-P600 and BDA-P700 CMSM and pore size distribution, respectively. As the carbonization temperature increases from 600 to 700 °C, CO_2_ sorption capacity increases, see Figure S2a, along with pore volume, from 0.13 to 0.16 cm^3^/g, and surface area, from 481.5 to 591.8 m^2^/g. This phenomenon can be explained by an increase in ultramicropores caused by shrinkage of the micropores [37]. Additionally, a notable shift in the pore size distribution towards smaller ultramicropores was observed, see Figure S2b [38]. As has been previously reported in the literature, elevated carbonization temperatures result in a more compact carbon structure and smaller pore sizes, which effectively exclude larger gas molecules (N_2_, CH_4_) and enhance the molecular sieving performance that would be expected from BDA-P600 and BDA-P700 CMSM.

### 3.6. X-Ray Photoelectron Spectroscopy (XPS)

**Figure 6:**
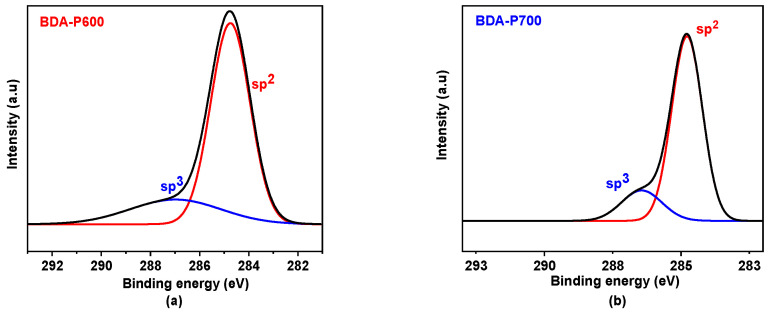
BDA-P600 (**a**) and BDA-P700 (**b**) C1s XPS spectrum and their deconvolution to sp^3^ and sp^2^ hybridization.

The X-ray photoelectron spectroscopy (XPS) measurements for BDA-P600 and BDA-P700 are presented in Figure 6a and 6b, respectively. To elucidate the chemical hybridization of carbon in the CMSM, the data were deconvoluted into two fitting peaks using a model based on a set of Gaussian–Lorentzian functions. The peak at ~284.8 eV is associated with graphite-like sp^2^ carbon bonds, C-C, C-H, and C=C, which are essential for the formation of ultramicropores [11]. Furthermore, an additional peak at ~285.5 eV is observed, which corresponds to the sp^3^ hybridization of C-C in the aromatic rings [39]. In general, a high sp^2^/sp^3^ ratio is indicative of a greater prevalence of sp^2^ carbon in the structure.

Table 4 presents a summary of the sp^2^/sp^3^ ratios calculated for CMSM-based BDA membranes. For BDA-P600 and BDA-P700, the ratio is 78 to 82%, which corresponds to sp^2^ hybridization, while approximately 20% corresponds to sp^3^ hybridization. However, BDA-P700 exhibits a significantly higher degree of graphitization (30%) compared to BDA-P600.

### 3.7. Raman Spectroscopy 

**Figure 7:**
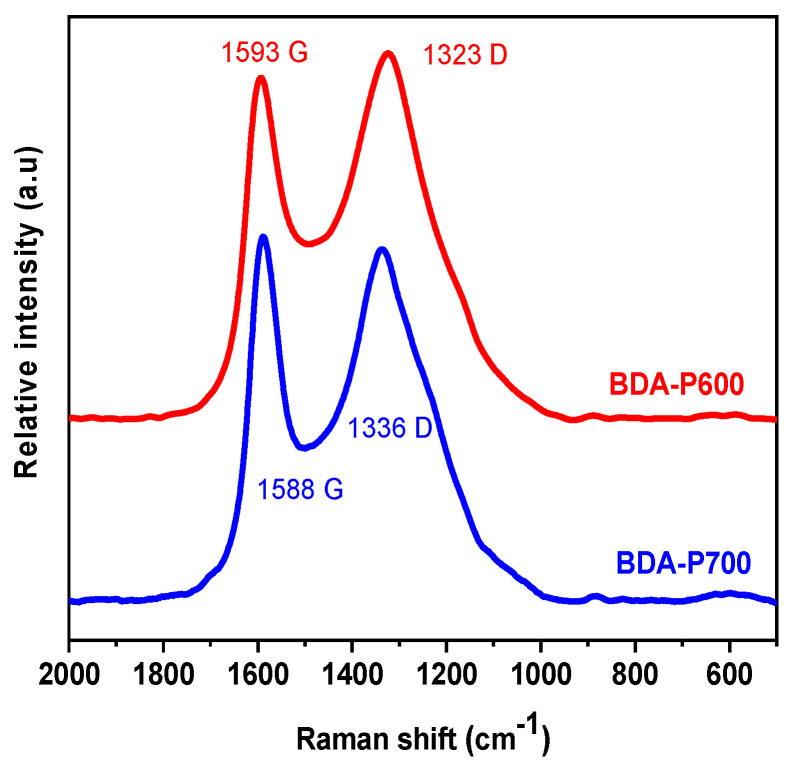
BDA-P600 and BDA-P700 CMSM Raman spectra showing G and D peaks.

The Raman spectrum represents another widely utilized characterization tool for carbon structures. BDA-P600 and BDA-P700 Raman spectra are presented in Figure 7. As observed, BDA-P600 and BDA-P700 membranes present a typical bimodal distribution, exhibiting two first-order bands in the region of 500–200 cm^−1^. The first, designated G (~1590 cm^−1^), is associated with the vibration of sp^2^ carbon atoms of E_2g_ symmetry, occurring in both ring and chain structures. The second band is associated with defect bands (~1340 cm^−1^), which are related to sp^2^ carbon atoms in the ring and to disordered graphite defects in CMSM [13]. A detailed analysis can be conducted by deconvoluting the main peaks into two characteristic bands using a Gaussian–Lorentzian function model, Figure S3a,b. Furthermore, the I_D/G_ ratio is associated with the graphitization level; a lower I_D/G_ ratio indicates a reduction in defects in the generated CMSM [40]. Table 5 presents a summary of the I_D/G_ ratios calculated for BDA-P600 and BDA-P700. It can be observed that for BDA-P600 and BDA-P700 a similar level of graphitization is present. The BDA-P600 and BDA-P700 Raman results demonstrate that the pyrolytic processing of BDA membranes at 600 °C and 700 °C results in the formation of similar carbonaceous structures, which is also consistent with the XPS results.

### 3.8. Pure Gas Permeability Coefficients in BDA Precursor Membrane, BDA-P600, and BDA-P700

Table 6 summarizes the gas permeability coefficients for the BDA precursor membrane, as well as those of BDA-P600 and BDA-P700, for the pure gases oxygen (O_2_), nitrogen (N_2_), methane (CH_4_), and carbon dioxide (CO_2_). The gas permeability coefficients’ observed behavior follows a typical trend, with CO_2_ exhibiting the highest permeability, followed by O_2_, CH_4_, and N_2_, consistent with the gas kinetic diameter. For BDA, pure gas permeability coefficients decrease in the sequence: *P*(CO_2_) > *P*(O_2_) > *P*(CH_4_) > *P*(N_2_) [4]. Additionally, for the gas pairs O_2_/N_2_, CO_2_/CH_4_, and N_2_/CH_4_, a clear tendency of increasing ideal gas selectivity for BDA-P600 and BDA-P700 was observed as pyrolysis temperature increased. BDA-P700’s ideal selectivity for O_2_/N_2_ is two times larger than that of BDA, while for CO_2_/CH_4_ and N_2_/CH_4_ the increase is almost three times. This increase in selectivity is attributed to a decrease in the permeability of CH_4_, while all the other gases show increases in permeability coefficients for BDA-P600 and BDA-P700 compared to BDA.

Table 7 provides a summary of the apparent diffusion coefficient (*D*) and the diffusion selectivity of BDA precursor membranes, BDA-P600, and BDA-P700. The order of diffusion coefficients is as follows: *D*(O_2_) > *D*(CO_2_) > *D*(N_2_) > *D*(CH_4_) [41]. In general, the diffusion coefficients increase with a rising pyrolysis temperature for BDA-P600 and BDA-P700. The apparent diffusion coefficients for O_2_ and N_2_—gases with low condensability—show only slight changes, while the more condensable gases—CH_4_ and CO_2_—exhibit a tendency to decrease with an increasing pyrolytic temperature. This behavior has been attributed to the formation of new micropores which become compacted as the pyrolysis temperature increases.

BDA-P600 exhibited a threefold increase in permeability (*P*) with respect to the BDA precursor for all gases tested. The ideal selectivity for CO_2_/CH_4_ increased 1.3-times, while for the remaining gas pairs a decrease was observed, as previously reported [12]. In contrast, BDA-P700 results in a slight decrease in permeability relative to BDA-P600. A detailed examination of Table 6 reveals that in the presence of O_2_, N_2_, and CH_4_ gases, the *P* value decreased twofold while the selectivity exhibited a notable increase. For instance, the ideal selectivity of α CO_2_/CH_4_ increased from 39.7 to 82.0 and from 6.4 to 11.3 for α O_2_/N_2_ for BDA-P600 and BDA-P700, respectively. This phenomenon can be attributed to two factors. Firstly, the denser packing of the crystals is evidenced by the *d*002 value (3.42 Å) for the BDA-P700 membranes. Secondly, the appearance of a new peak in the WAXD result 2θ = 43.6° (2.1 Å) effectively reduced the diffusion of these gases (O_2_, N_2_, and CH_4_). In this context, Xu et al. [33] observed the presence of ultramicropores in poly(arylene ether ketone) (PEKC) within a range similar to that detected in the BDA polymer at ~2θ = 45°. This was directly linked to a reduction in the (002) peak and an increase in the estimated crystallite stacking thickness (*L_c_*) and layer plane size (*L_a_*), indicating the formation of more ordered graphitic structures. These findings align closely with the results reported in this study.

**Figure 8:**
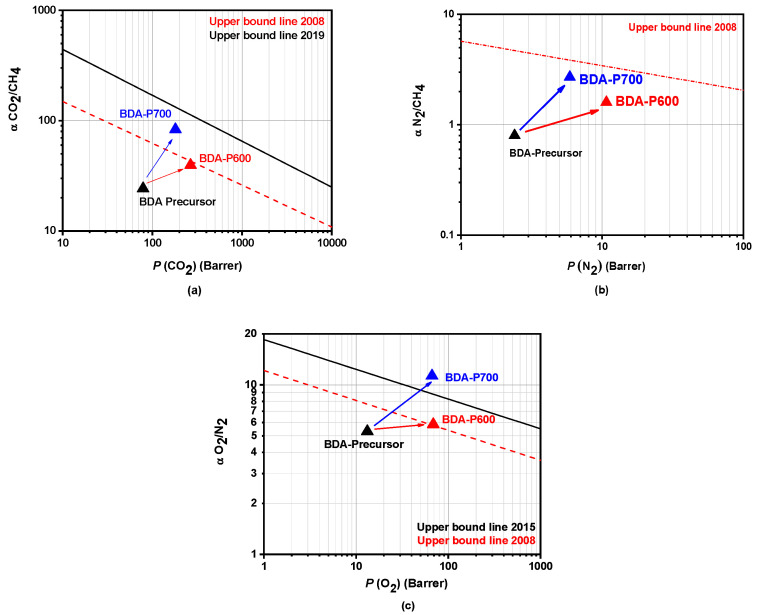
Robeson plot separation performance for gas pairs (**a**) CO_2_/CH_4_, (**b**) N_2_/CH_4_, and (**c**) O_2_/N_2_ for BDA-P600, BDA-P700, and BDA precursor at 35 °C and 2 atm.

The overall CO_2_/CH_4_, N_2_/CH_4_, and O_2_/N_2_ BDA precursor ideal gas selectivity and those found for BDA-P600 and BDA-P700 are shown in Figure 8. For reference, the upper bounds proposed by Robeson in 2008 [42], 2015, and 2019 [43] are also included. After pyrolysis, N_2_ permeability decreased significantly from 10.7 to 2.2 Barrer, CH_4_ from 6.7 to 2.2 Barrer, and CO_2_ from 266.1 to 180.1 Barrer for BDA-P600 and BDA-P700, respectively. At the same time, gas pair selectivity improved substantially: CO_2_/CH_4_ increased 2-fold, N_2_/CH_4_ increased 1.7-times, and O_2_/N_2_ increased 1.76-fold (Figure 8a–c). These changes shifted performance from well below the 2008 upper bound lines [42] to significantly above the 2008 trade-off curves, approaching the latest 2015 and 2019 upper bound limits [43].

### 3.9. Temperature Test: Effect on Pure Gas Permeability Coefficients in BDA Polymer Dense Membranes, BDA-P600, and BDA-P700

**Figure 9:**
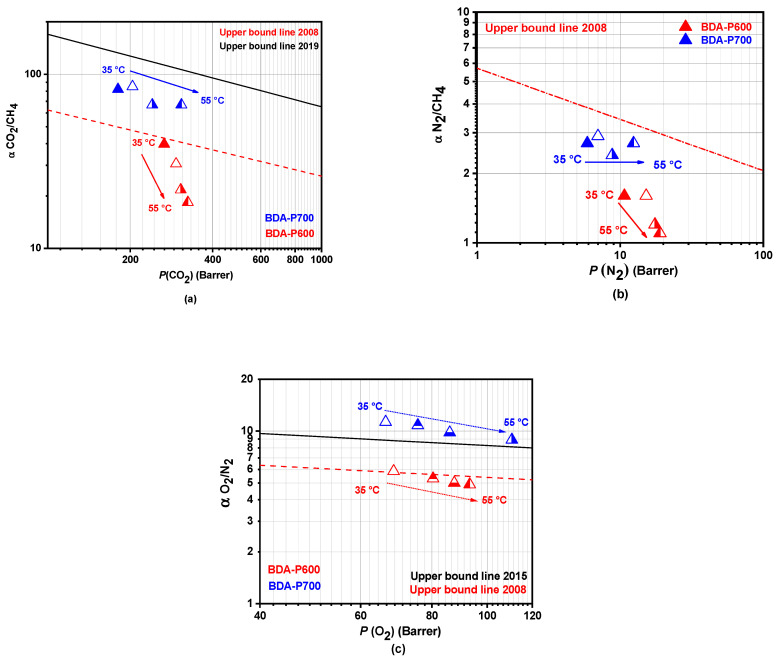
Effect of test temperature between 35 and 55 °C on ideal separation factors for (**a**) CO_2_/CH_4_, (**b**) N_2_/CH_4_, and (**c**) O_2_/N_2_ gas pairs for BDA-P600 and BDA-P700 CMSM (gas permeation tests carried out with a 2 atm feed pressure).

Sanyal et al. [44] emphasize that the gas separation performance is influenced not only by the feed pressure but also significantly by the test temperature, which affects both the gas permeability and selectivity. As illustrated in Figure 9, BDA-P600 (red triangle) shows a 26.3% and 18% enhancement in O_2_ and CO_2_ permeabilities, respectively, when the test temperature increases from 35 to 55 °C. However, there was a notable decline in selectivity for O_2_/N_2_ with a 23% reduction, while a more pronounced selectivity decrease, 53.6%, for the CO_2_/CH_4_ gas pair was observed. Similarly, BDA-P700 (blue triangle) exhibited approximately a 1.6-fold increase in *P*(O_2_) and *P*(CO_2_), while ideal gas selectivity decreased by 21% for O_2_/N_2_ and 18.5% for CO_2_/CH_4_. It is noteworthy that, despite the decline in permeability for BDA-P700 with an increasing test temperature, their performance exceeded the 2015 trade-off limit proposed by Comesaña et al. [43]. A summary of the gas transport properties with increasing temperature for BDA-P600 and BDA-P700 CMSM is provided in Tables S1 and S2.

### 3.10. CMSM Apparent Sorption Coefficient and Gas Condensability

The solubility of gases in membranes can be influenced by several factors, including their boiling point (T_*b*_), critical temperature (T_*c*_), and the Lennard-Jones temperature parameter (*ϵ*/*k*). These properties can be linked to variations in gas solubility through the following equation: ln S_A_ = *M* + *N*(*ϵA*/*k*), where M and N are fitting parameters. The parameter *M* depends on polymer–penetrant interactions and can fluctuate significantly between polymers, while *N* is reported to remain nearly constant at approximately 0.023 K^−1^ for both glassy and rubbery polymers [45]. Table 8 summarizes the solubility coefficient S for the BDA precursor and the sorption coefficient *S* for BDA-P600 and BDA-P700. For the gas pairs O_2_/N_2_ and CO_2_/CH_4_, the solubility selectivity for the BDA precursor shows a trend in agreement with what was expected; however, for CO_2_/N_2_ the value is relatively high compared to other polymeric membranes [46].

BDA-P600 and BDA-P700 show higher sorption coefficients, with values above those for BDA. As the pyrolysis temperature increases, the sorption coefficients for BDA-P600 and BDA-P700 also rise. A closer examination of Table 8, which outlines the sorption coefficients for highly soluble gases such as CO_2_ and CH_4_, shows a significant increase compared to the precursor membranes. However, sorption selectivity drops sharply to approximately 0.07. These results were correlated with a decrease in the (002) peak and the simultaneous appearance of peak 100, associated with the crystallite stacking thickness.

**Figure 10:**
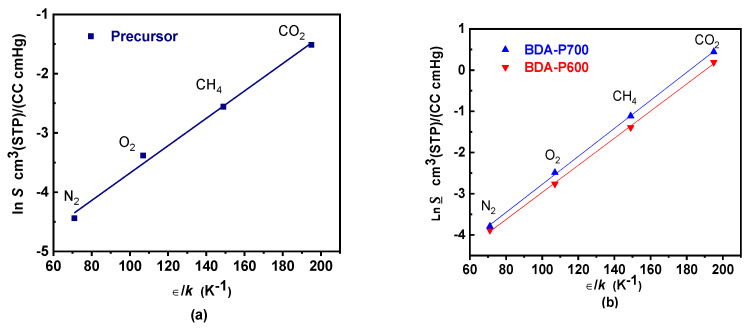
Gas solubility coefficient S in (**a**) BDA precursor and gas sorption coefficient *S* in (**b**) BDA-P600 and BDA-P700 CMSM as a function of the Lennard-Jones temperature parameter (ϵ/k) for gases N_2_, O_2_, CH_4_, and CO_2_.

**Figure 11:**
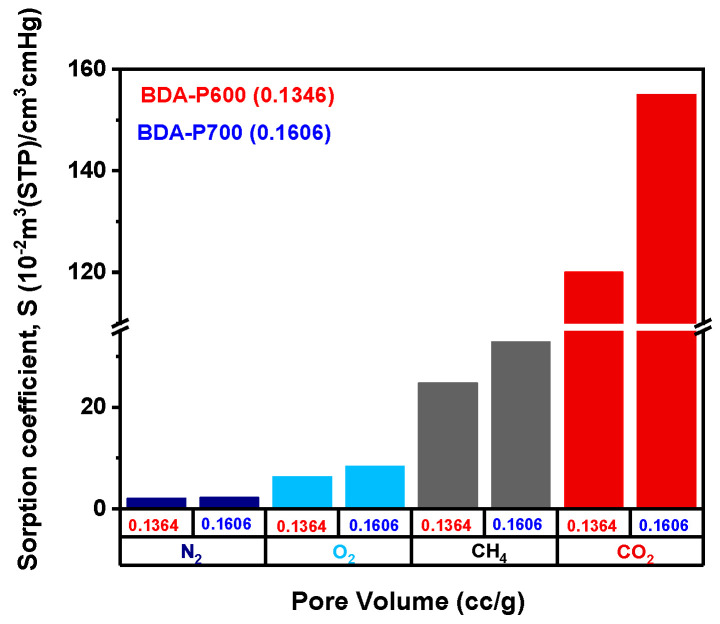
Gas sorption coefficient *S* for N_2_, O_2_, CH_4_, and CO_2_ as pore volume changes for BDA-P600 and BDA-P700.

Gas solubility (or sorption) coefficients are plotted in Figure 10a for BDA, and in Figure 10b for BDA-P600 and BDA-P700, as a function of the Lennard-Jones temperature (ε/k). Table 9 summarizes the *N* and *M* parameter values found using linear regression for ln S vs. (ε/k) and ln *S* vs. (ε/k). For BDA, *N* = 0.0231 and *M* = −5.989 are close to those expected for glassy polymers as reported elsewhere [47]. On the other hand, for BDA-P600 and BDA-P700 there is a change in *N*, increasing to 0.032 and 0.033, respectively. This is an indication of increasing gas sorption due to the larger surface area and pore volume available in BDA-P700 as compared to BDA-P600 due to a rearrangement of the carbonaceous graphitic structure. Figure 11 summarizes the *S* behavior for BDA-P600 and BDA-P700. As a result of a larger pore volume, BDA-P700 *S* increases by approximately 15% with respect to BDA-P600; consequently, the gas sorption coefficient increases by 22.5%. This result is also due to a surface area increase of 18.6% for BDA-P700 as compared to BDA-P600. As a result, there is an increase in gas sorption of at least 10% even for low condensability gases such as N_2_ and O_2_, while CO_2_ increases by 30%.

Analysis of the CMSM gas sorption results indicate that the observed increase in gas sorption can be attributed to two factors. Firstly, the presence of a larger average carbon layer spacing within the carbon structure, and secondly, the presence of a higher pore volume and surface area created during the pyrolysis process. Therefore, it can be concluded that the increase in gas sorption is linked to the differences in pore volume microstructure and gas condensability in BDA-P600 and BDA-P700 CMSM.

## 4. Conclusions

The synthesis of polymer (1,1′-biphenyl)-6,8a-dihydroacenaphthylene-1(2H)-one (BDA) from acenaphthenequinone was successfully accomplished. The BDA polymer’s high decomposition temperature (T_d_ = 620 °C) and char yield, 85 wt%, renders it a viable candidate for CMSM formation. Pyrolyzing BDA precursors at 600 °C, BDA-P600, promotes the formation of ultramicropores, resulting in a carbon structure with more open spacing between graphene-like strands in a carbonaceous amorphous matrix. This structural change is evidenced by an increase in the apparent crystalline thickness (*L_c_*) and the apparent layer plane length (*L_a_*). When the pyrolysis temperature is raised to 700 °C, BDA-P700, the average stacking number of carbon layers, *N*, also increases, which leads to further increases in *L_c_* and *L_a_*. Additionally, the WAXD results show the emergence of a new peak at 2θ = 43.6° (2.1 Å), which effectively hinders O_2_, N_2_, and CH_4_ diffusion. This behavior has been attributed to the formation of new micropores that become increasingly compact at higher pyrolysis temperatures. The CO_2_ sorption results at 273 °K indicate that the BDS-P700 pore volume and surface area are larger than those of BDA-P600. The increase in porosity and surface area results in a higher gas sorption for all gases in BDA-P700. As a result, BDA-P700 CMSM derived from BDA precursors pyrolyzed at 700 °C demonstrates exceptional gas separation performance, particularly for O_2_/N_2_, significantly surpassing baseline trade-off limits.

## Figures and Tables

**Table 1:** BDA polymer solubility in chlorinated, ketone, and aprotic solvents.

Solvent	Polymer BDA
Dichloromethane	−
Sym-tetrachloroethane	+
Chloroform	−
H_2_SO_4_	−
Dimethylformamide	−
N-methyl-2-pyrrolidone	+
Tetrahydrofurane	−
Dimethyl sulfoxide	−
Dimethylacetamide	−
Pyridine	−
Ciclohexanone	−
1,4-Dioxane	−

**Table 2:** WAXD spectrum deconvolution results for BDA precursor, BDA-P600, and BDA-P700.

Deconvolution	Peak 1			Peak 2			Peak 3			r^2^
	Xc ^(a)^	W ^(b)^	A ^(c)^	Xc	W	A	Xc	W	A	
Precursor	5.70	1.40	0.12	11.0	2.1	0.91	13.67	12.94	10.38	0.987
BDA-P600	25.49	14.79	15.79	11.8	6.8	3.57	40.35	6.98	1.22	0.989
BDA-P700	25.95	9.20	7.4	43.4	4.7	0.49	12.47	8.81	1.78	0.956

^(a)^ Peak position. ^(b)^ Peak width. ^(c)^ Peak area.

**Table 3:** The structural parameters of graphitic crystallites in BDA-P600 and BDA-P700 obtained by the WAXD analysis.

Sample	2θ (002)	*d*002 (Å) ^a^	FWHM (002) (°)	2θ (100)	*L_a_*(Å) ^b^	*L_c_*(Å) ^c^	N ^d^
BDA-P600	25.49	3.49	14.79	40.35	24.78	5.75	2.64
BDA-P700	25.95	3.42	9.25	43.46	36.95	9.20	3.69

^a^ *d*002 is calculated from the (002) peak using Bragg’s law (*d*002= λ/2sinθ, where λ = 1.54 Å). ^b,c^ *L_a_* and *L_c_* are calculated using Scherrer’s equation (*L_a_*/c = Kλ/β cosθ; *L_c_* is calculated from (002) peak, where K = 0.89 and β is the FWHM of the (002) peak; *L_a_* is calculated from the (100) peak, where K = 1.84, and β is the FWHM of the (100) peak). ^d^ N is the average stacking number of carbon layers, which is calculated from N = *L_c_*/*d*002 +1.

**Table 4:** BDA-P600 and BDA-P700 membranes’ degree of graphitization from Cs1 in XPS spectrum.

Polymer	C-C sp^2^	C-C sp^3^	Graphitization Degree
	(%)	(%)	
BDA-P600	77.9	22.1	3.52
BDA-P700	82.2	17.8	4.61

**Table 5:** Gaussian decomposition data from BDA-P600 and BDA-P700 Raman spectra.

Polymer/Signal	Intensity	
	BDA-P600	BDA-P700
G	78.4	86.0
D	218.0	236.1
I_D/G_	2.78	2.74

**Table 6:** Pure gas permeability coefficients and ideal separation performance at 35 °C and 2 atm for BDA precursor membrane, BDA-P600, and BDA-P700 CMSM.

Sample	Permeability (Barrer) ^a^				Ideal Selectivity		
	O_2_	N_2_	CH_4_	CO_2_	α O_2_/N_2_	α CO_2_/CH_4_	α N_2_/CH_4_
Precursor	13.2	2.4	3.2	78.6	5.3	24.5	0.8
BDA-P600	68.5	10.7	6.7	266.1	6.4	39.7	1.6
BDA-P700	66.4	5.9	2.2	180.5	11.3	82.0	2.7

^a^ 1 Barrer = 1 × 10^−10^ cm^3^ (STP) cm/cm^2^ s cm Hg. All data were measured three times each with +/− 2% error.

**Table 7:** Apparent gas diffusion coefficients for BDA precursor membrane, BDA-P600, and BDA-P700 CMSM.

Sample	Diffusion Coefficient (10^−8^ cm^2^/s)				Diffusion Selectivity (*D*_A_/*D*_B_)		
	O_2_	N_2_	CH_4_	CO_2_	O_2_/N_2_	CO_2_/CH_4_	CO_2_/N_2_
precursor	6.9	2.1	0.43	3.1	3.3	7.2	1.5
BDA-P600	10.9	5.2	0.27	2.2	2.1	8.1	0.42
BDA-P700	7.9	2.6	0.07	1.2	3.1	16.5	0.45

**Table 8:** Apparent solubility coefficient, S, for BDA precursor membrane and apparent sorption coefficients, *S*, for 600 and 700 °C pyrolyzed based BDA CMSM.

Sample	Solubility Coefficient, S, (10^−2^ cm^3^ (STP)/cm^3^ cm Hg)				Solubility Selectivity (*S_A_*/*S_B_*)		
	O_2_	N_2_	CH_4_	CO_2_	O_2_/N_2_	CO_2_/CH_4_	CO_2_/N_2_
Precursor	1.91	1.18	7.5	25	1.0	2.8	11.8
**Sample**	**Sorption Coefficient, S (10^−2^ m^3^ (STP)/cm^3^ cm Hg)**				**Sorption Selectivity (*S_A_*/*S_B_*)**		
	**O_2_**	**N_2_**	**CH_4_**	**CO_2_**	**O_2_/N_2_**	**CO_2_/CH_4_**	**CO_2_/N_2_**
BDA-P600	6.3	2.0	24.8	120	3.0	0.08	0.21
BDA-P700	8.3	2.2	32.8	155	3.6	0.07	0.21

**Table 9:** Gas solubility coefficient constant correlation with the Lennard-Jones parameter (ϵ/k) for BDA polymeric membrane and gas sorption constants for BDA-P600 and BDA-P700 CMSM.

Sample	$N\;(\in_{A}/k)$	M ^a^	r^2^
BDA	0.0231	−5.989	0.997
BDA-P600	0.0329	−6.256	0.999
BDA-P700	0.0339	−6.170	0.999

^a^ Units are cm^3^ (STP)/(CC cm Hg).

## Data Availability

The original contributions presented in this study are included in the article/Supplementary Material. Further inquiries can be directed to the corresponding authors.

## Supplementary Materials

The following supporting information can be downloaded at: https://www.mdpi.com/article/10.3390/polym17040541/s1; Figure S1. WAXD spectrum deconvolution of BDA Precursor, BDA-P600 and BDA-P700; Figure S2. BDA-P600 and BDA-P700 (a) CO2 sorption isotherms at 0 °C (b) corresponding pore volume and surface area of pores smaller than 7 Å estimated using the NLDFT method; Figure S3. Raman spectrum deconvolution (a) BDA-P600 and (b) BDA-P700; Table S1. BDA-P 600 CMSM gas Permeability coefficients and gas separation performance between 35 and 55 °C and 2 atm; Table S2. BDA-P700 CMSM gas permeability coefficients and gas separation performance between 35 to 55 °C and 2 atm.

## Abbreviations

TFSA	Trifluoromethanesulfonic acid
TFA	Trifluoroacetic acid
MeOH	Methanol
CH_2_Cl_2_	Methylene chloride
MSA	Methanesulfonic acid
NMP	1-methyl-2-pyrrolidone
BDA	poly (biphenyl-6, 8-dihydroacenaphthalenyl.-1-ona)
DCM	dichloromethane
CHCl_3_	Chloroform
TCE	1,1,2,2-tetrachloroethane
THF	tetrahydrofuran
DMAc	N,N-Dimethylacetamide
DMF	N,N-dimethylformamide
DMSO	Dimethyl sulfoxide
H_2_SO_4_	Sulfuric acid
υ	Specific volume
υ_w_	Van der Waals volume
FVV	Fractional free volume
CMC	Carbon molecular sieve membrane
TCE-d_2_	Deuterated tetrachloroethane
XPS	X-ray Photoelectron Spectroscopy
TGA	Thermal Gravimetric Analysis
NMR	Nuclear Magnetic Resonance
FTIR	Fourier transform infrared
WAXD	Wide angle X-Ray
*P*	Pure gas permeability coefficients
*D*	Apparent diffusion coefficient
*S*	Apparent solubility coefficient
*S*	Apparent gas sorption
*L_a_*	apparent layer-plane length
*L_c_*	apparent crystallite thickness
Å	Ángstrom
FWHM	Full width at half maximum
I_D/G_	Level of graphitization
Td	Decomposition temperature
T*_b_*	Boiling point
T*_c_*	Critical temperature
ε/k	correlation with Lennard-Jones temperature

## References

[1] Wang M., Zhao J., Wang X., Liu A., Gleason K.K. (2017). Recent Progress on Submicron Gas-Selective Polymeric Membranes. J. Mater. Chem. A Mater..

[2] Bernardo P., Drioli E., Golemme G. (2009). Membrane Gas Separation: A Review/State of the Art. Ind. Eng. Chem. Res..

[3] Jusoh N., Yeong Y.F., Lau K.K., Azmi M.S. (2015). Membranes for Gas Separation Current Development and Challenges. Appl. Mech. Mater..

[4] Sharif A. (2018). Polymeric Gas Separation Membranes: What Makes Them Industrially More Attractive?. J. Membr. Sci. Res..

[5] Tanco M.A.L., Tanaka D.A.P. (2016). Recent Advances on Carbon Molecular Sieve Membranes (CMSMs) and Reactors. Processes.

[6] Zhou W., Yoshino M., Kita H., Okamoto K.I. (2001). Carbon Molecular Sieve Membranes Derived from Phenolic Resin with a Pendant Sulfonic Acid Group. Ind. Eng. Chem. Res..

[7] Kim Y.K., Park H.B., Lee Y.M. (2003). Carbon Molecular Sieve Membranes Derived from Metal-Substituted Sulfonated Polyimide and Their Gas Separation Properties. J. Membr. Sci..

[8] Salleh W.N.W., Ismail A.F. (2011). Carbon Hollow Fiber Membranes Derived from PEI/PVP for Gas Separation. Sep. Purif. Technol..

[9] Jiao W., Ban Y., Shi Z., Jiang X., Li Y., Yang W. (2017). Gas Separation Performance of Supported Carbon Molecular Sieve Membranes Based on Soluble Polybenzimidazole. J. Membr. Sci..

[10] Araújo T., Bernardo G., Mendes A. (2020). Cellulose-Based Carbon Molecular Sieve Membranes for Gas Separation: A Review. Molecules.

[11] Deng M., Wei J., Du W., Qin Z., Zhang Z., Yang L., Yao L., Jiang W., Tang B., Ma X. (2024). High-Performance Carbon Molecular Sieve Membranes Derived from a PPA-Cross-Linked Polyimide Precursor for Gas Separation. ACS Appl. Mater. Interfaces.

[12] Hu C.P., Polintan C.K., Tayo L.L., Chou S.C., Tsai H.A., Hung W.S., Hu C.C., Lee K.R., Lai J.Y. (2019). The Gas Separation Performance Adjustment of Carbon Molecular Sieve Membrane Depending on the Chain Rigidity and Free Volume Characteristic of the Polymeric Precursor. Carbon N. Y..

[13] Li K., Zhu Z., Dong H., Li Q., Ji W., Li J., Cheng B., Ma X. (2021). Bottom up Approach to Study the Gas Separation Properties of PIM-PIs and Its Derived CMSMs by Isomer Monomers. J. Membr. Sci..

[14] Sanyal O., Zhang C., Wenz G.B., Fu S., Bhuwania N., Xu L., Rungta M., Koros W.J. (2018). Next Generation Membranes—Using Tailored Carbon. Carbon N. Y..

[15] Ortiz-Espinoza J., Loría-Bastarrachea M.I., Hernández-Cruz O., Aguilar-Lugo C., de Jesus Montes-Luna Á., Zolotukhin M., Ruiz-Treviño F.A., Aguilar-Vega M. (2024). Carbon Molecular Sieve Membranes from Multiring Highly Aromatic Functional Copolymers. ACS Appl. Polym. Mater..

[16] Ortiz-Espinoza J., Cetina-Mancilla E., Zolotukhin M.G., Ruiz-Treviño F.A., Baas-López J.M., Sulub-Sulub R., Loría-Bastarrachea M.I., Aguilar-Vega M.J. (2024). Carbon Molecular Sieve Membranes from Poly(Oxo-Biphenylene-Isatin) with Increasingly Bulky Fluorine Substitution: Characterization and Gas Transport Properties. Ind. Eng. Chem. Res..

[17] Fu Y.J., Liao K.S., Hu C.C., Lee K.R., Lai J.Y. (2011). Development and Characterization of Micropores in Carbon Molecular Sieve Membrane for Gas Separation. Microporous Mesoporous Mater..

[18] (2010). Test Method for Density of Plastics by the Density-Gradient Technique.

[19] Wu A.X., Lin S., Mizrahi Rodriguez K., Benedetti F.M., Joo T., Grosz A.F., Storme K.R., Roy N., Syar D., Smith Z.P. (2021). Revisiting Group Contribution Theory for Estimating Fractional Free Volume of Microporous Polymer Membranes. J. Membr. Sci..

[20] Wang Q., Huang F., Cornelius C.J., Fan Y. (2021). Carbon Molecular Sieve Membranes Derived from Crosslinkable Polyimides for CO_2_/CH_4_ and C_2_H_4_/C_2_H_6_ Separations. J. Membr. Sci..

[21] Wollbrink A., Volgmann K., Koch J., Kanthasamy K., Tegenkamp C., Li Y., Richter H., Kämnitz S., Steinbach F., Feldhoff A. (2016). Amorphous, Turbostratic and Crystalline Carbon Membranes with Hydrogen Selectivity. Carbon N. Y..

[22] Eusébio T.M., Martins A.R., Pon G., Faria M., Morgado P., Pinto M.L., Filipe E.J.M., de Pinho M.N. (2020). Sorption/Diffusion Contributions to the Gas Permeation Properties of Bi-Soft Segment Polyurethane/Polycaprolactone Membranes for Membrane Blood Oxygenators. Membranes.

[23] Amer A.M., Askar S.I., Muhdi T.S. (2014). Reactions of acenaphthenequinone derivatives with some aromatic and aliphatic amines. Eur. Chem. Bull..

[24] Klumpp D.A., Zhang Y., Do D., Kartika R. (2008). Reactions of Acenaphthenequinone and Aceanthrenequinone with Arenes in Superacid. Appl. Catal. A Gen..

[25] Zolotukhin M.G., Fomina L., Salcedo R., Sansores L.E., Colquhoun H.M., Khalilov L.M. (2004). Superelectrophiles in Polymer Chemistry. A Novel, One-Pot Synthesis of High-Tg, High-Temperature Polymers. Macromolecules.

[26] Zolotukhin M.G., Fomine S., Lazo L.M., Salcedo R., Sansores L.E., Cedillo G.G., Colquhoun H.M., Fernandez-G J.M., Khalizov A.F. (2005). Superacid-Catalyzed Polycondensation of Acenaphthenequinone with Aromatic Hydrocarbons. Macromolecules.

[27] Cruz A.R., Hernandez M.C.G., Guzmán-Gutiérrez M.T., Zolotukhin M.G., Fomine S., Morales S.L., Kricheldorf H., Wilks E.S., Cárdenas J., Salmón M. (2012). Precision Synthesis of Narrow Polydispersity, Ultrahigh Molecular Weight Linear Aromatic Polymers by A2 + B2 Nonstoichiometric Step-Selective Polymerization. Macromolecules.

[28] Cruz-Rosado A., Romero-Hernández J.E., Rios-López M., López-Morales S., Cedillo G., Rios-Ruiz L.M., Cetina-Mancilla E., Palacios-Alquisira J., Zolotukhin M.G., Vivaldo-Lima E. (2023). Non-Stoichiometric Effect in the Superacid-Catalyzed Polyhydroxyalkylation of Biphenyl and 1-Propyl Isatin. High Perform. Polym..

[29] Guzmán-Gutiérrez M.T., Nieto D.R., Fomine S., Morales S.L., Zolotukhin M.G., Hernandez M.C.G., Kricheldorf H., Wilks E.S. (2011). Dramatic Enhancement of Superacid-Catalyzed Polyhydroxyalkylation Reactions. Macromolecules.

[30] Qiu J., Albrecht J., Janey J. (2020). Solubility Behaviors and Correlations of Common Organic Solvents. Org. Process Res. Dev..

[31] Jiang M., Liu Q., Zhang Q., Ye C., Zhou G. (2012). A Series of Furan-Aromatic Polyesters Synthesized via Direct Esterification Method Based on Renewable Resources. J. Polym. Sci. A Polym. Chem..

[32] Rungta M., Wenz G.B., Zhang C., Xu L., Qiu W., Adams J.S., Koros W.J. (2017). Carbon Molecular Sieve Structure Development and Membrane Performance Relationships. Carbon N. Y..

[33] Xu R., He L., Li L., Hou M., Wang Y., Zhang B., Liang C., Wang T. (2020). Ultraselective Carbon Molecular Sieve Membrane for Hydrogen Purification. J. Energy Chem..

[34] Yoon Y.H., O’Nolan D., Beauvais M.L., Chapman K.W., Lively R.P. (2023). Direct Evidence of the Ultramicroporous Structure of Carbon Molecular Sieves. Carbon N. Y..

[35] Monshi A., Foroughi M.R., Monshi M.R. (2012). Modified Scherrer Equation to Estimate More Accurately Nano-Crystallite Size Using XRD. World J. Nano Sci. Eng..

[36] Lei L., Pan F., Lindbråthen A., Zhang X., Hillestad M., Nie Y., Bai L., He X., Guiver M.D. (2021). Carbon Hollow Fiber Membranes for a Molecular Sieve with Precise-Cutoff Ultramicropores for Superior Hydrogen Separation. Nat. Commun..

[37] Smith R.L. (2023). Stacking Disorder as a Critical Tuning Parameter for the Properties of Materials. Ph.D. Thesis.

[38] Ye C., Luo C., Ji W., Weng Y., Li J., Yi S., Ma X. (2023). Significantly Enhanced Gas Separation Properties of Microporous Membranes by Precisely Tailoring Their Ultra-Microporosity through Bromination/Debromination. Chem. Eng. J..

[39] Cockroft S.L., Hunter C.A., Lawson K.R., Perkins J., Urch C.J. (2005). Electrostatic Control of Aromatic Stacking Interactions. J. Am. Chem. Soc..

[40] Cao Y., Zhang K., Sanyal O., Koros W.J. (2019). Carbon Molecular Sieve Membrane Preparation by Economical Coating and Pyrolysis of Porous Polymer Hollow Fibers. Angew. Chem..

[41] Sharififar A., Bakhtiari O., Bayati B. (2023). Molecular Dynamics Simulation of Gas Permeability Through Polyvinyl Acetate Membrane. J. Membr. Sci. Res..

[42] Robeson L.M. (2008). The Upper Bound Revisited. J. Membr. Sci..

[43] Comesaña-Gándara B., Chen J., Bezzu C.G., Carta M., Rose I., Ferrari M.C., Esposito E., Fuoco A., Jansen J.C., McKeown N.B. (2019). Redefining the Robeson Upper Bounds for CO_2_/CH_4_ and CO_2_/N_2_ Separations Using a Series of Ultrapermeable Benzotriptycene-Based Polymers of Intrinsic Microporosity. Energy Environ. Sci..

[44] Sanyal O., Hays S.S., León N.E., Guta Y.A., Itta A.K., Lively R.P., Koros W.J. (2020). A Self-Consistent Model for Sorption and Transport in Polyimide-Derived Carbon Molecular Sieve Gas Separation Membranes. Angew. Chem. Int. Ed..

[45] Xie H., Simha R. (1997). Theory of Solubility of Gases in Polymers. Polym. Int..

[46] Najafia P., Penchahb H.R., Ghaemia A. (2020). Improving CO_2_/N_2_ and CO_2_/H_2_ selectivity of Hypercrosslinked Carbazole-Based Polymeric Adsorbent for Environmental Protection. J. Chem. Pet. Eng..

[47] Freeman B.D., Pinnau I. (1999). Polymeric Materials for Gas Separations. Polymeric Membranes for Gas and Vapor Separations.

