# Supplementary material for: Carbon Molecular Sieve Membranes from Acenaphthenequinone–Biphenyl Polymer; Synthesis, Characterization, and Effect on Gas Separation and Transport Properties
Source: Polymers (Basel). 2025 Feb 19;17(4):541. doi: 10.3390/polym17040541 (PMC11859076; doi:10.3390/polym17040541)
Supplement: Supplementary file 1 [file polymers-17-00541-s001.zip › polymers-3446866-supplementary.pdf]

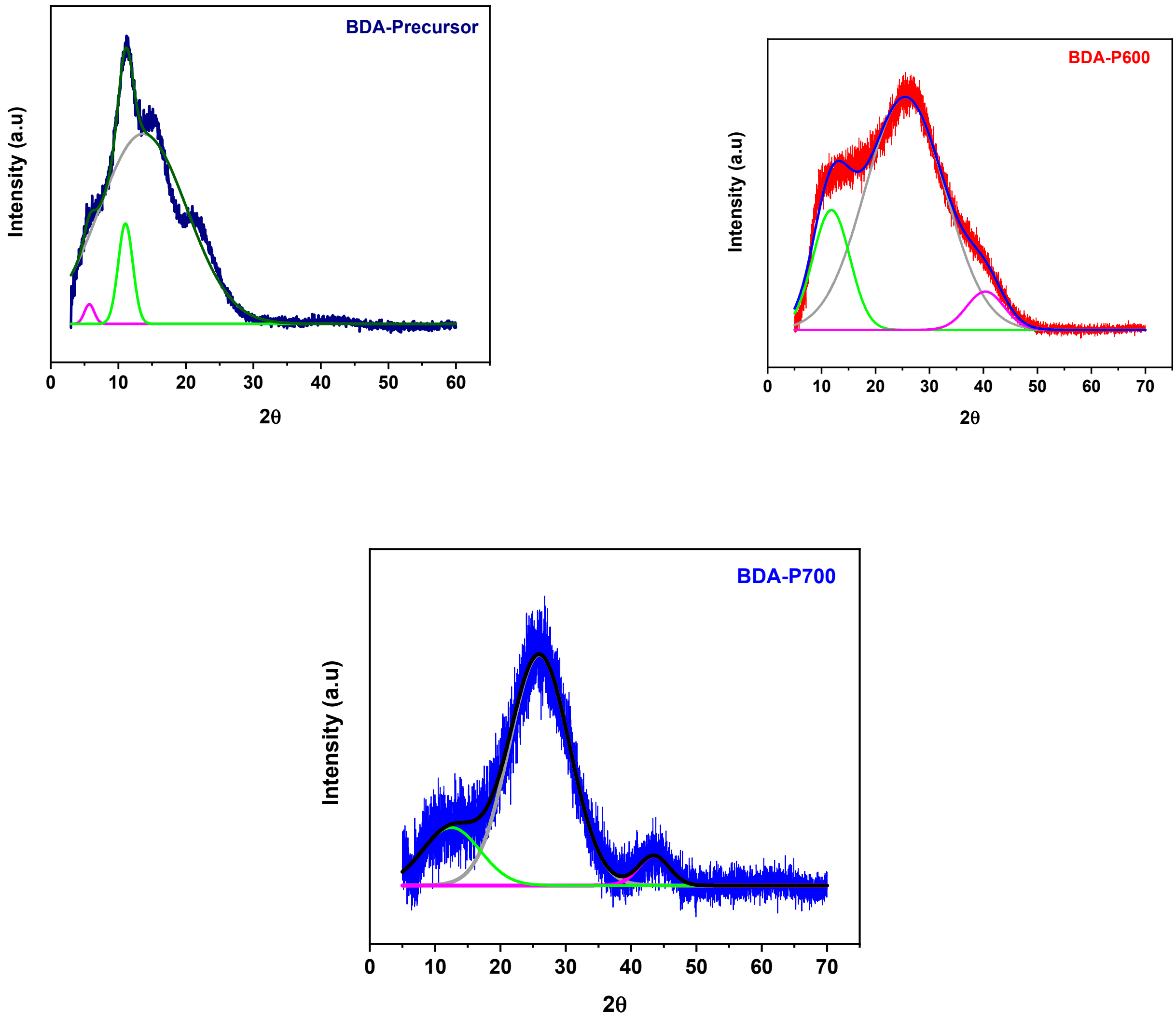

Figure S1. WAXD spectrum deconvolution of BDA Precursor, BDA-P600 and BDA-P700.

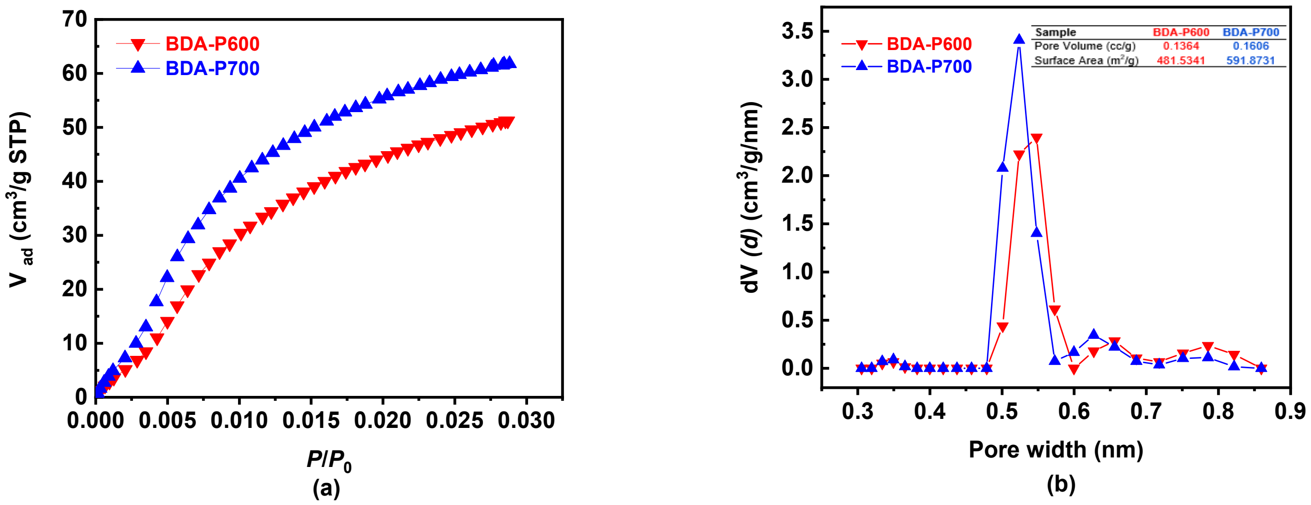

Figure S2. BDA-P600 and BDA-P700 (a) CO_2 sorption isotherms at 0ºC (b) corresponding pore volume and surface area of pores smaller than 7 Å estimated using the NLDFT method.

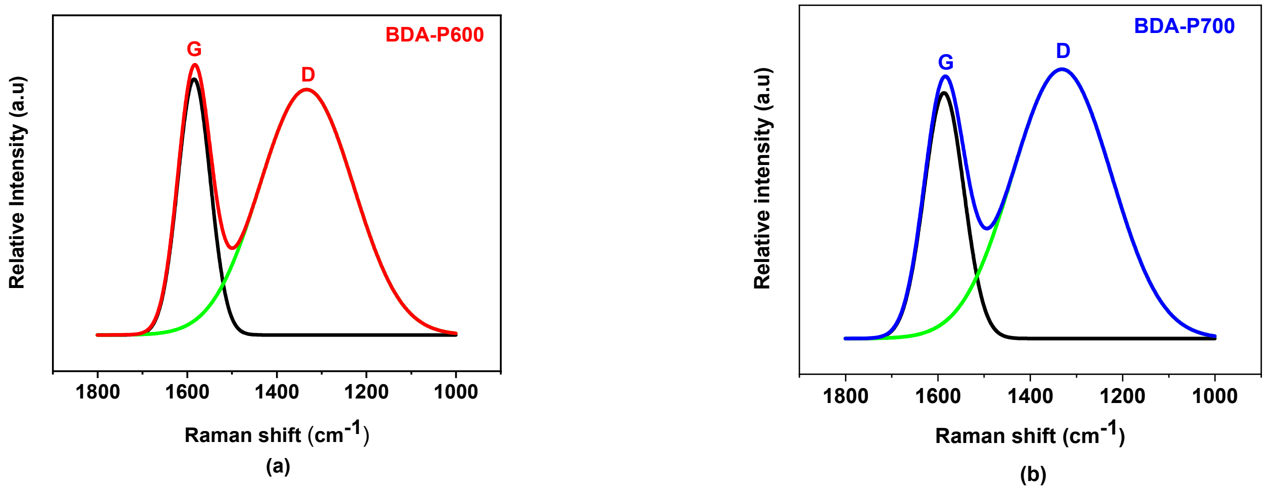

Figure S3. Raman spectrum deconvolution (a) BDA-P600 and (b) BDA-P700.

Table S1. BDA-P 600 CMSM gas Permeability coefficients and gas separation performance between 35 and 55°C and 2 atm.

BDA-P600	Permeability (Barrer)				Ideal Selectivity (α)			
(T °C)	O_2	N_2	CH_4	CO_2	O_2/N_2	CO_2/CH_4	CO_2/N_2	N_2/CH_4
**35**	68.6	10.7	6.7	266.1	6.4	39.7	25.0	1.6
**40**	80.4	15.2	9.6	294.1	5.3	30.6	19.3	1.6
**45**	87.6	17.5	14.1	306.0	5.0	21.7	17.5	1.2
**55**	93.2	19.0	17.6	324.7	4.9	18.4	17.1	1.1

Table S2. BDA-P700 CMSM gas permeability coefficients and gas separation performance between 35 to 55°C and 2 atm

BDA-P700	Permeability (Barrer)				Ideal Selectivity(α)			
T(°C)	O_2	N_2	CH_4	CO_2	O_2/N_2	CO_2/CH_4	CO_2/N_2	N_2/CH_4
**35**	66.4	5.9	2.2	180.5	11.3	82.0	30.6	2.7
**40**	75.6	7.0	2.4	203.9	10.8	85.0	29.1	2.9
**45**	86.0	8.8	3.6	240.7	9.8	66.8	27.3	2.4
**55**	110.5	12.4	4.6	307.5	8.9	66.8	24.8	2.7
